# Electrochemical detection of NGF using a reduced graphene oxide- titanium nitride nanocomposite

**DOI:** 10.1038/s41598-018-25196-z

**Published:** 2018-05-02

**Authors:** Zheng Wei, Yanchun Wang, Junping Zhang

**Affiliations:** 1 Department of Oncology, Henan Academy Institute of Traditional Chinese Medicine, Zhengzhou, 450004 Henan China; 2 Department of Traditional Chinese Medicine, Henan Province People Hospital, Zhengzhou, 450004 Henan China

## Abstract

There is a correlation between the severity of neurological impairment in patients that have suffered a cerebrovascular accident and the nerve growth factor (NGF) level. This study addressed the fabrication of a titanium nitride (TiN) and reduced graphene oxide (RGO)-based composite with remarkable electrocatalytic activity towards NGF oxidation in a phosphate buffer solution (PB, 0.1 M). The proposed electrochemical sensor was linearly related to the NGF concentration in the range of 10 nM-5 μM with a detection limit of 2.6 nM.

## Introduction

The cortical and brainstem areas are engaged in micturition control^1^. Cerebrovascular accidents (CVAs) lead to transient detrusor underactivity (DUA) in the acute phase, reduced bladder capacity and uninhibited detrusor contractions in the following phase, and stabilization in the resolution phase^2^. More serious micturition abnormalities have been detected in patients suffering cerebral atrophy, multiple infarcts and bilateral lesions^3^. Furthermore, 49% of patients had difficulty with voiding and suffered nocturnal urinary frequency and urinary retention within the initial 3 months after experiencing an acute brainstem stroke^4^. There are three main symptoms of patients suffering acute hemisphere stroke, namely, nocturnal urinary frequency, urge urinary incontinence (UUI) and voiding difficulty; these symptoms were present in 36%, 29% and 25% of patients^4^, respectively. Common urodynamic manifestations in CVA patients include uninhibited sphincter relaxation, detrusor sphincter dyssynergia (DSD) and detrusor overactivity (DO). A remarkable reduction was observed in the bladder capacity of cerebral-infarcted rats during animal experiments, while cerebral-infarcted rats and sham-operated rats exhibited no obvious distinction in their contractile response of detrusor strips and bladder weight^5^. In another study of cerebral-infarcted rats, an increase in the expression of the neural plasticity-related gene was observed in their pontine tegmental area^6^. The activation of the bladder afferents and the modulation of the motor neuron activity in CVA were indicated from the inhibition of DO, and the reduction in the expression of c-fos & zif268 mRNA was caused by N-methyl-d-aspartate (a glutamatergic receptor antagonist)-engaged pretreatment. The dysregulation of the brain instead of that of the visceral motor units was found to lead to cerebral infarction-induced DO, as indicated in the aforementioned results. There are no phasic detrusor contractions observed at the filling stage during urodynamic studies in patients suffering CVA, where uninhibited contraction is observed at bladder capacity. After receiving antimuscarinic agent-involved therapy, the patients suffering overactive bladder syndrome (OAB) gain a reduced perception of urgency severity as well as an increased bladder capacity^7^. Nevertheless, only partial control can be clinically achieved for CVA-induced DO by antimuscarinics. The motor neuron activity also has the potential to be mediated by the significant mechanoreceptors on visceral afferents with respect to the CVA-induced DO. Nonetheless, more severe brain damage, undesirable prognosis and elevated mortality rates at 1 year after the stroke onset are suggested by a subtype of UI after stroke, where an impaired awareness of UI (IA-UI) is observed^8,9^.

The smooth muscle and urothelium in the urinary tract produce nerve growth factor (NGF)^10^. OAB, interstitial cystitis and other lower urinary tract dysfunctions are found to be directly related to increased levels of NGF in the urine and bladder tissue, according to the clinical and experimental results^11–13^. NGF has been found to be mainly produced by the visceral epithelia, and the function of adult motor and sensory neurons are assumed to be regulated by NGF^14^. The patients suffering sensory urgency and DO have shown increased NGF levels in their urine and bladder tissue^15–17^.

Generally, the detection and quantification of haptens, infectious agents, DNA, proteins, antibodies and other substances are addressed via enzyme-linked immunosorbent assay (ELISA) techniques under immunological reactions. Due to advantages in accuracy, sensitivity, and straightforwardness as well as the relative low cost and suitability for automation and large-scale specimen analysis, ELISA methods have gained significant popularity^18^. Nevertheless, a time-consuming procedure is routinely needed for ELISA techniques. Fortunately, a significantly sensitive, selective, facile and cost-effective method of electrochemical detection has recently been achieved.

Graphene, composed of a single-atom-thick sheet of hexagonal lattice bonded sp^2^ carbon atoms is recognized among the most exciting carbon nanomaterials. Due to the high theoretical surface area and advantages in optical, mechanical, thermal and electronic features, it has gained great attention^19–42^. In addition, the distinct features of metal nitrides make them appealing to many researchers, where the transition metal nitrides are regarded as having more potential for diverse applications than other recently researched metal nitrides due to their physicochemical features^43,44^. Herein, titanium nitride (TiN) with desirable corrosion stability, oxidation resistance and conductivity^45^ is regarded to be remarkable in fields such as solar cells^46^, supercapacitors^47^, lithium-ion batteries^48^, and biosensors^49–53^. Hence, improved electrochemical behavior could be obtained through the integration of TiN and graphene with outstanding features.

This work addressed the electrochemical determination of NGF via a reduced graphene oxide (RGO)–TiN nanocomposite synthesized by thermal nitridation of a RGO–titanium dioxide (TiO_2_) composite. In addition to its remarkable long-term stability and reproducibility, the obtained composite showed desirable sensitivity with a low limit of detection (LOD) for NGF and exhibited a desirable recovery in the determination of NGF levels, which made it feasible for applications in biomedical fields.

## Experiments

### Chemicals

Titanium (IV) isobutoxide (Ti(OBu)_4_) and graphite were commercially available from Sigma-Aldrich. All reagents were analytical grade, and the solutions were prepared with deionized water. Jinan Military General Hospital provided the nerve growth factor (NGF).

### Preparation of RGO–TiO_2_ nanocomposite and RGO–TiN nanocomposite

This work employed a modified Hummers’ technique to prepare graphene oxide (GO)^54^. During the composite preparation, a GO dispersion was obtained via the sonication of GO (1 g) in an ethanol solution (250 mL). Then, Ti(OBu)_4_ (10 mL) was added to the obtained dispersion and left stirring for 1 h. This step was followed by the addition of deionized water (5 mL) and continuous stirring for another 1 h. Subsequently, the as-prepared mixture was refluxed for 6 h to obtain a TiO_2_ precursor-coated GO surface, which was then washed by deionized water and ethanol. Eventually, the RGO*–*TiO_2_ nanocomposite was synthesized after the sample was annealed for 120 min at 200 °C under N_2_ and heated at 500 °C in a furnace. For the water and air to be removed, N_2_ was purged in the aforementioned furnace for 20 min prior to heating. Then, NH_3_ (100 cm^3^/min) was purged as soon as the temperature rose to 500 °C. The thermal nitridation process continued for 120 min. After the furnace was cooled via N_2_ purging, the final RGO*–*TiN nanocomposite product was obtained.

### Electrochemical determination

The electrochemical carbendazim detection was performed with a three-electrode configuration, where the counter and reference electrodes were respectively a Pt foil and a saturated calomel electrode (SCE). Cyclic voltammetry (CV) was performed at scan rate of 50 mV/s. Differential pulse voltammetry (DPV) measurements were carried out with an amplitude, pulse width, sampling width and pulse period of 30 mV, 0.02 sec, 0.015 sec and 0.05 sec, respectively. In addition, electrochemical impedance spectroscopy (EIS) was carried out in PB (0.1 M) containing Fe(CN)_6_^3−/4−^ (5 mM) at ambient temperature.

### Characterization

Al Kα radiation was used in the X-ray photoelectron spectroscopy (XPS, Thermo Scientific, K–Alpha) analysis to determine the surface features. A Raman microscope (WITEC alpha 300R) provided the platform for Raman spectroscopy with an excitation wavelength of 532 nm. A Cu Kα radiation source was applied in an X-ray diffractometer (XRD, X’Pert PRO MRD, Philips) to record the sample crystallinity.

## Results and Discussion

**Figure 1:**
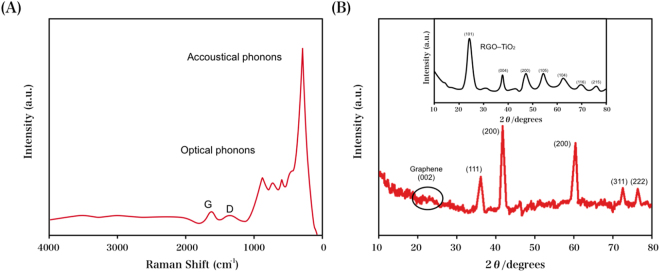
(**A**) Raman spectrum and (**B**) XRD profiles of the RGO–TiN nanocomposite.

The RGO–TiN nanocomposite synthesis was realized via a two-step process. This process began with the synthesis of the RGO–TiO_2_ nanocomposite via coating the TiO_2_ precursor onto the surface of the GO and annealing in the presence of N_2_ for 120 min at 200 °C. This was followed by heating for 120 min under NH_3_ at 800 °C to obtain the RGO–TiN composite, which was characterized via Raman spectroscopy, as shown in Fig. 1A. Herein, the D band and G band were respectively observed at 1356 and 1601 cm^−1^, with the former corresponding to the breathing mode of the rings and the latter corresponding to the sp^2^ hybridized carbon atoms. Furthermore, the Raman scattering of TiN was indicated by the bands at 130, 404, 516, and 633 cm^−1^. The RGO–TiN and RGO–TiO_2_ nanocomposites were characterized via the XRD profiles in Fig. 1B, with the characteristic peaks of the anatase crystal structure of TiO_2_ observed for the latter^55^. The face-centered cubic structure (JCPDS No.) of TiN was observed with the (111), (200), (220), (222) and (311) reflections denoted by diffraction peaks at 37.3, 43.2, 62.6, 79.2, and 75.0°, respectively. Additionally, the (002) reflection of RGO was suggested by the peak at 2θ = 25.0°^56–58^. Herein, the RGO–TiN composite was successfully obtained, as confirmed by the aforementioned results.

**Figure 2:**
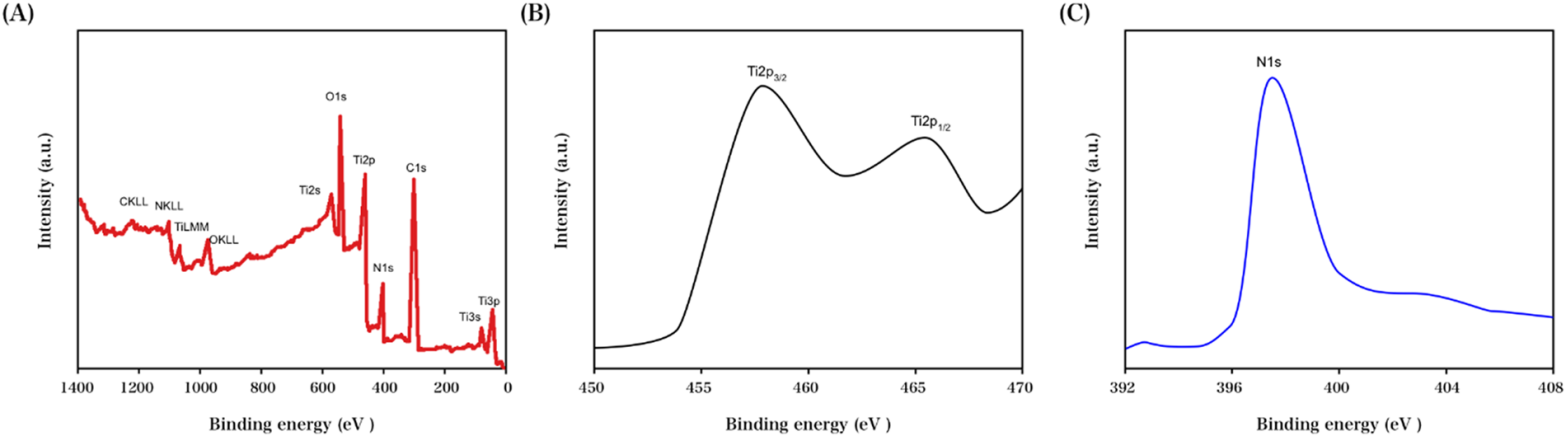
(**A**) XPS survey, (**B**) individual Ti 2p spectrum, and (**C**) core-level N 1 s spectrum of the RGO–TiN nanocomposite.

As indicated in Fig. 2A, Ti_3s_ (59.7 eV), Ti_3p_ (33.2), Ti_2s_ (565.2), Ti_2p_ (456.7), N_1s_ (395.5), O_1s_ (529.3) and C_1s_ (285.1) were displayed in the survey spectrum of the RGO–TiN composite, and the successful modification of TiN on the surface of RGO was confirmed. The 463.5 and 458.2 peaks exhibited in the separated Ti 2p spectrum (Fig. 2B) respectively corresponded to the binding energies of Ti_2p1/2_ and Ti_2p3/2_. Compared with the Ti^4+^ peaks, the aforementioned peaks shifted to comparatively lower binding energies. Figure 2C indicates the survey spectrum of N 1 s.

**Figure 3:**
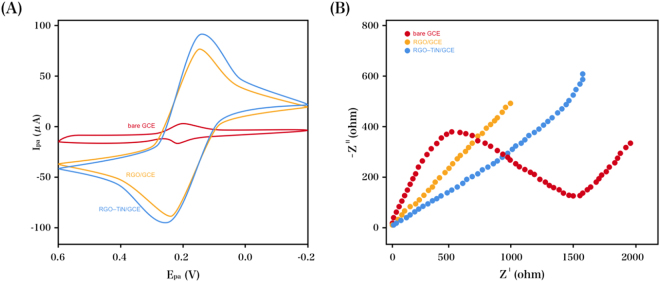
(**A**) CVs and (**B**) Nyquist plots of the original GCE, RGO/GCE and RGO–TiN/GCE in 5 mM Fe(CN)_6_^3−/4−^ (1:1) solution containing KCl (0.1 M).

Electrochemical impedance spectroscopy (EIS) and cyclic voltammetry (CV) were employed for the characterization of the as-prepared electrode in an [Fe(CN)_6_]^3−/4−^ solution (5 mM) containing KCl (0.1 M). The most insubstantial voltammetric response was observed at the original electrode (Fig. 3A). Compared with the original GCE, RGO–TiN/GCE and RGO/GCE exhibited significantly larger background and peak currents. It is believed that the effective active area of electrodes was enhanced by the RGO–TiN or RGO composite films, and the electron exchange rate was accelerated by the conductive properties of the RGO–TiN or RGO components. As indicated in Fig. 3B, the as-modified electrodes were characterized via Nyquist plots. A semicircle region and a linear region were shown in the impedance spectroscopy, where the former denoted the charge transfer-limited process (*R*_ct_) and the latter suggested the diffusion-limited process^59,60^. The greatest impedance was obtained at the GCE. Due to the enhanced electron exchange efficiency from RGO, there was a great decrease in the impedance of the RGO-modified GCE. Compared with the RGO/GCE, the RGO–TiN/GCE exhibited a comparatively smaller semicircle due to the electrical conductivity of TiN.

**Figure 4:**
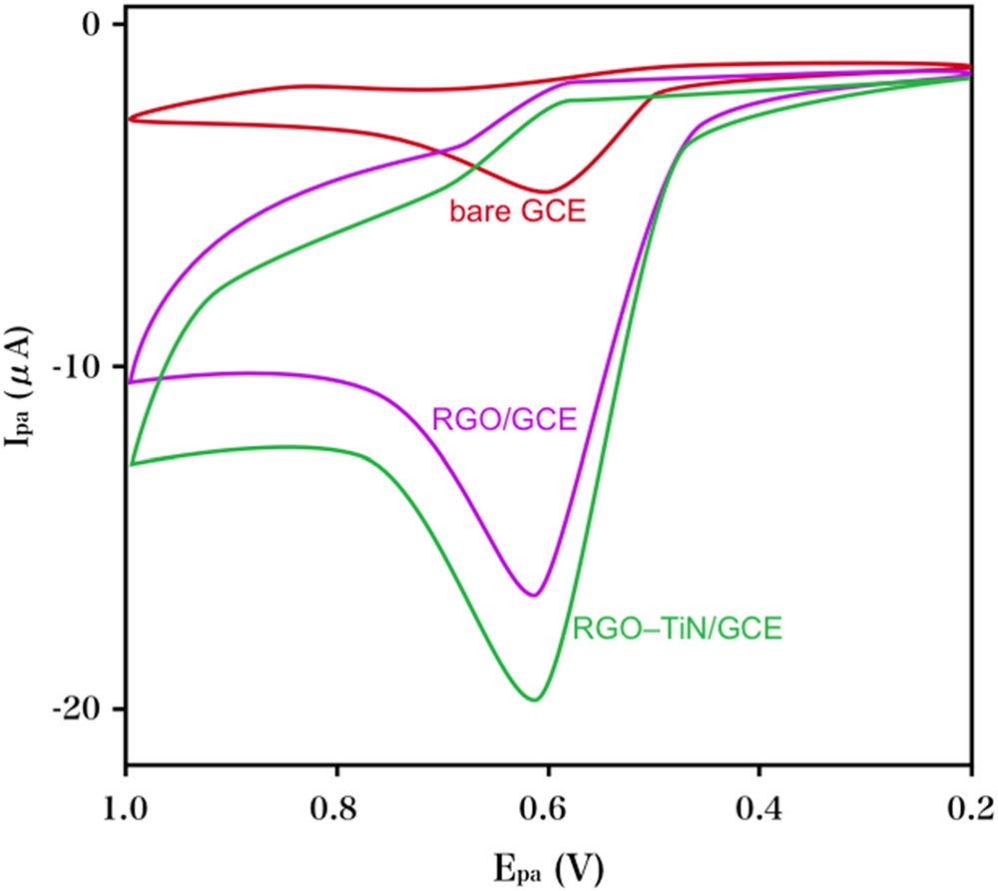
CV profiles of bare GCE, RGO/GCE and RGO- TiN/GCE towards NGF (0.1 mM) in PB (0.1 M) with a pH of 8.0 at a scan rate of 50 mV/s.

The performance of NGF (0.1 mM) on varying electrodes in PB (0.1 M) at a pH of 8.0 was studied via CV. The lowest oxidative signal was observed at the original GCE, according to Fig. 4. There was a remarkable increase in the oxidative peak current of the RGO-coated GCE, which suggested the capacity of the RGO film towards improving the electrochemical oxidization to NGF. Even more significant oxidative responses were exhibited by RGO–TiN/GCE than RGO/GCE, which could possibly be attributed to the remarkable promotion of the NGF-GCE electron exchange through the greater conductivity of TiN.

**Figure 5:**
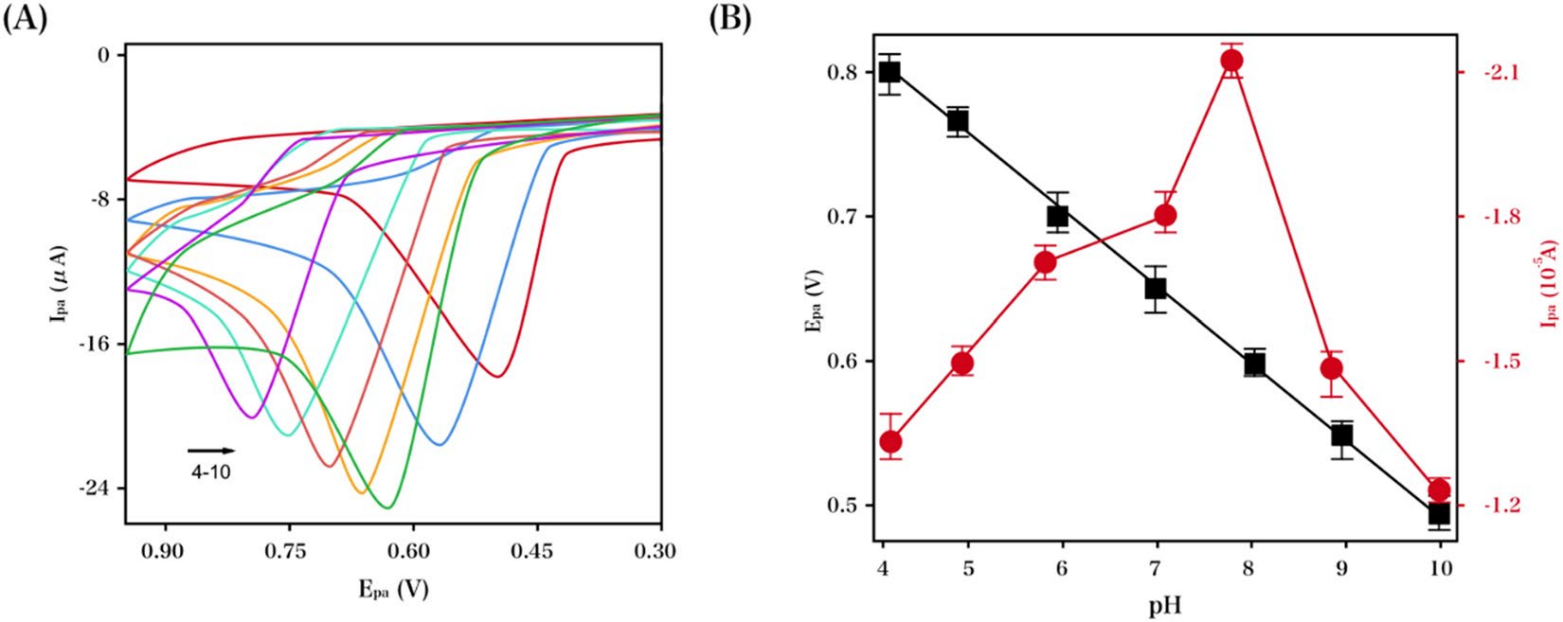
(**A**) CV profiles of 0.1 mM NGF at RGO–TiN/GCE in PB (0.1 M) with varying pH values. (**B**) Influence of the pH on the peak current and potential at a scan rate of 50 mV/s.

The CV responses of NGF in PB with varying pH values were displayed, with the influence of the latter on the former studied herein. NGF levels in PB (0.1 M) over a pH range of 4–10 at RGO–TiN/GCE was characterized via CVs in Fig. 5A. As indicated in 5B, the maximal oxidative peak current of NGF was obtained at a pH of 8.0 in the aforementioned pH range; hence the supporting PB electrolyte was set at a pH of 8 to obtain the desirable sensitivity. Nevertheless, there was a proportional shift in the negative direction with respect to the oxidation peak potential, with a linear equation of the peak potential (*E*_pa_) vs. pH presented as *E*_pa_ (V) = −0.0517 pH + 1.0288 (*R* = 0.99). With a slope (51.7 mV/pH) in the vicinity of the theoretical Nernstian value (−58 mV/pH), it could be suggested that an equivalent number of protons and electrons are engaged in the electro-oxidation pathway of NGF at the RGO–TiN/GCE.

**Figure 6:**
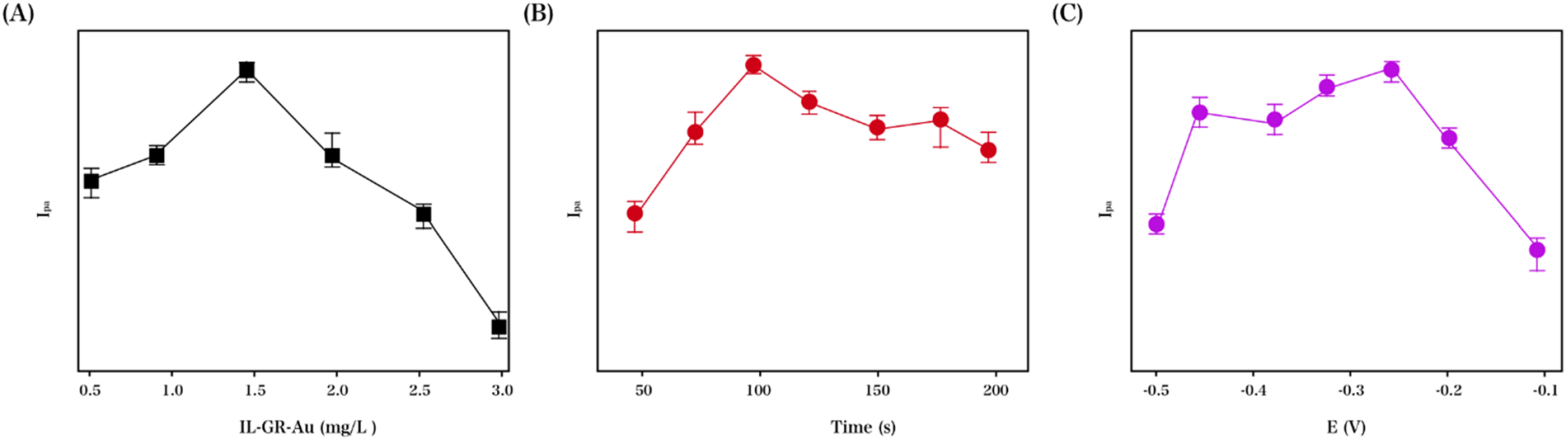
Effects of (**A**) RGO–TiN content, (**B**) accumulation time and (**C**) accumulation potential on the oxidation peak current of NGF (0.1 mM) in PB (0.1 M) with a pH of 8.0.

This work also emphasized the optimization of the influence of the accumulation potential and time as well as the RGO–TiN loading to achieve more desirable sensitivity. The peak current of NGF rose to a maximal value at 1.5 mg/mL with a concentration range of 0.5–3.0 mg/mL with respect to the RGO–TiN/GCE suspension content, as indicated in Fig. 6A. In addition, Fig. 6B addresses the accumulation time factor, in which an unremittent increase in the oxidation current peak response before 100 s of accumulation was observed, followed by an insignificant decrease and subsequent stable state. Hence, this experiment set the accumulation time at 100 s. The accumulation potential was characterized in Fig. 6C, where a maximal oxidation peak was observed at −0.25 V with a potential window of −0.1 to −0.5 V, and thus, −0.25 V was determined as the accumulation potential in the following experiments.

**Figure 7:**
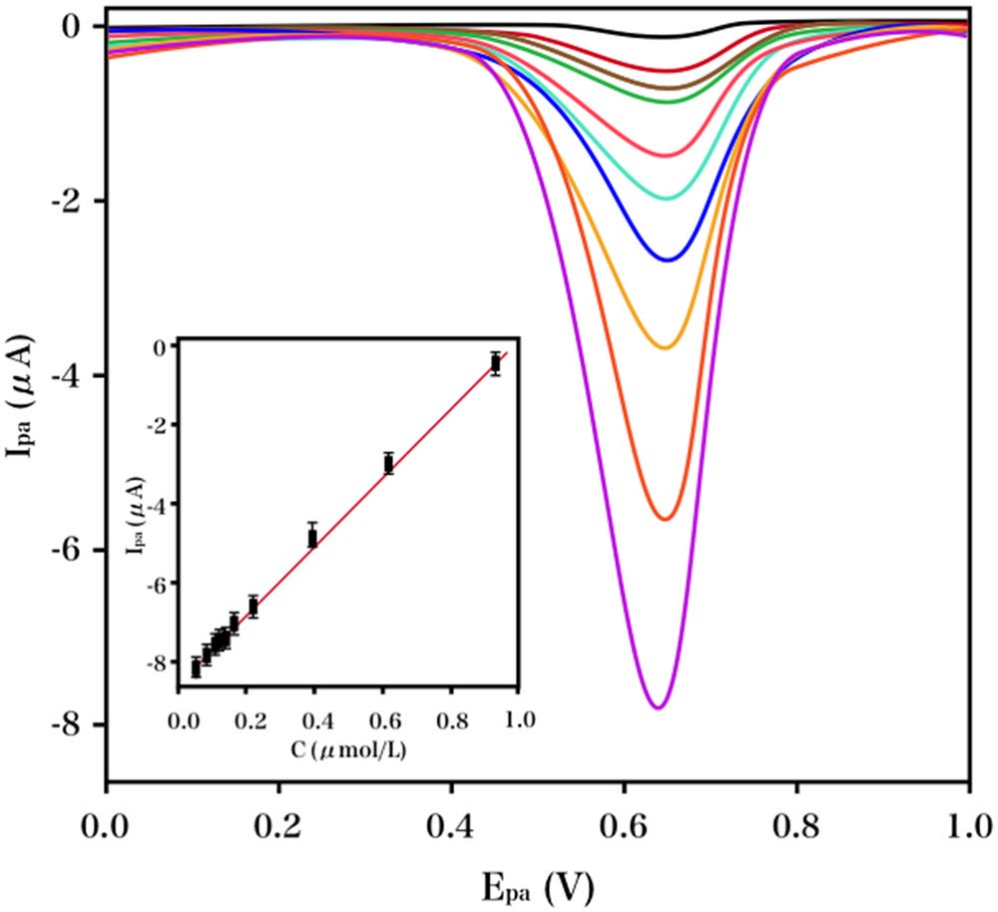
DPV profiles of NGF at RGO–TiN/GCE in PB (0.1 M, pH 8.0) containing varying concentrations of NGF. Insert: linear calibration curve.

The electrochemical response of NGF at an RGO–TiN/GCE with varying concentrations was detected via differential pulse voltammetry (DPV), which was more sensitive than CV. As the concentration of NGF was increased over the extensive range of 10 nM-5 μM, there was a linear increase in the oxidation peak current (Fig. 7), with an LOD of 2.6 nM (S/N = 3). The increased active sites, extensive surface area and excellent conductivity were the major contributing factors for the broad linear range and low LOD.

The sensing behavior was assessed via the measurement of the shelf life, reproducibility and stability of the as-prepared RGO–TiN/GCE, where 10 DPV scans were applied to the electrode for stability. This electrode was confirmed to have a desirable stability, as suggested by a peak current decrease of only 7.4%. NGF determination was conducted to measure the ten composite electrodes prepared separately for the assessment of reproducibility, with a relative standard deviation (RSD) of 3.31%. Herein, the obtained sensor was confirmed desirably reproducible to NGF. The detection of NGF in the presence of the same concentrations of acetylsalicylic acid, bacitracin zinc and ibuprofen showed negligible current changes. The effects of some common inorganic ions, such as Na^+^, K^+^, Cl^−^, CH_3_COO^−^ and CO_3_^2−^, were investigated. Most of the ions did not significantly interfere with the determination, except for CH_3_COO^−^. The presence of a 20-fold excess of CH_3_COO^−^ caused a change in the detection current of approximately 7%.

NGF detection in urine specimens was performed with as-prepared RGO–TiN/GCE to obtain real specimen detection. The specimens were diluted 10-fold with PB (0.1 M, pH = 7) prior to the determination. The concentration of spiked NGF was measured via a standard addition approach. Table 1 indicates the data with respect to the DPV measurement, with a range of desirable recoveries from 99.5 to 103.55%. Herein, the significantly sensitive and selective features of the as-prepared sensor were reflected by this range in carrying out successful NGF detection.

**Table 1:** Determination of NGF in urine samples (n = 3).

Real sample	NGF added (μM)	NGF found (μM)	RSD (%)	Recovery (%)
1	10	9.95	2.11	99.50
2	20	20.71	3.06	103.55
3	30	31.03	2.51	103.43

## Conclusions

This work fabricated an RGO–TiN nanocomposite for the electrochemical detection of NGF. The RGO sheets underlying the TiN nanoparticles provided good conductive supports, decreased aggregation of the TiN particles, and enhanced the electrochemical activity of TiN through synergistic chemical coupling effects. The DPV determination was linearly related to the concentration of NGF ranging from 10 nM to 5 μM with an LOD of 2.6 nM, which suggests a desirable sensitivity. Based on the real sample tests, the obtained composite has the potential to be effectively applied to the determination of NGF in biological samples.
